# Respiratory symptoms among Swedish soldiers after military service abroad: association with time spent in a desert environment

**DOI:** 10.1080/20018525.2017.1327761

**Published:** 2017-05-31

**Authors:** Johannes Saers, Linda Ekerljung, Bertil Forsberg, Christer Janson

**Affiliations:** ^a^ Department of Medical Sciences: Respiratory, Allergy and Sleep Research, Uppsala University, Uppsala, Sweden; ^b^ Department of Internal Medicine and Clinical Nutrition, Sahlgrenska Academy, University of Gothenburg, Gothenburg, Sweden; ^c^ Occupational and Environmental Medicine, Department of Public Health and Clinical Medicine, Umeå University, Umeå, Sweden

**Keywords:** Asthma, wheeze, cough, military, desert

## Abstract

**Introduction:** The aim of this paper was to study whether Swedish soldiers who have served abroad had a higher prevalence of respiratory symptoms than the general population and, if this was the case, also to study whether this was associated with time spent in a desert environment.

**Methods:**The prevalence of respiratory symptoms among 1,080 veterans from Kosovo and Afghanistan was compared with that in almost 27,000 subjects from a general population sample, using propensity score matching and logistic regression.

**Results:**The prevalence of wheeze (16.3 vs. 12.3%), wheeze without a cold (11.1 vs. 8.0%), nocturnal coughing (26.6 vs. 20.1%) and chronic bronchitis (12.3 vs. 6.8%) was significantly higher among soldiers than controls (*p* < 0.05). A dose-response-related association was found between time spent in a desert environment and wheeze, wheeze with breathlessness and wheeze when not having a cold. Having been exposed to desert storms was related to nocturnal cough and chronic bronchitis.

**Conclusion:**Swedish soldiers who had served abroad had a higher prevalence of wheeze and cough than a control group from the general population. The association between being exposed to a desert environment and respiratory symptoms indicates that further protective measures should be introduced for military personnel serving in a desert environment.

## Introduction

Since 1956, more than 100,000 Swedish soldiers have served abroad on various international missions. The increasing international involvement of state and non-state actors, during military operations, or civilian disaster relief and reconstruction, has put more and more personnel in new and challenging environments. There is a well-known connection between exposure to particulate matter (PM) air pollution and health effects.[1] Guidelines issued by the World Health Organisation for airborne particulate matter < 2.5 μm in diameter (PM 2.5) suggest that 25 μg m^–^^3^ is an acceptable level. Nevertheless, in countries such as Afghanistan, levels of up to 10,000 μg m^–^^3^ are regularly reported and levels can stay this high for prolonged periods of time.[2] The health effects caused by airborne particulate matter pollution including sandstorm dust [3,4] depend on the particle composition, which in turn depends on its source.[5] The different effects that PM has on pulmonary cells can be traced to the different biological and toxicological effects of different molecules in the PM.[6]

In a prospective study of American Armed Forces personnel, service men and women deployed to Iraq and Afghanistan had a higher rate of onset of respiratory symptoms compared with those not deployed. In addition, increased symptom reporting was linearly associated with increased deployment time.[7] British male soldiers deployed to the Gulf War in 1990–1991 had a higher prevalence of asthma and bronchitis than those deployed to Bosnia within the same timeframe.[8] An elevated rate of acute respiratory symptoms during deployment and a higher risk of post-deployment respiratory symptoms and illnesses were found in a review of 19 studies investigating respiratory health amongst military personnel deployed to Afghanistan and Iraq.[2] There is, however, a lack of data comparing the prevalence of respiratory symptoms in soldiers that have returned from missions abroad with that of the general population. Such studies are important in order to know if the reported negative effects on respiratory health is associated with military work in general or to specific exposures in subgroups. There is also a need for more investigations studying the possible association between respiratory health and the time spent in a desert environment.

The aim of this paper was to investigate whether Swedish soldiers who have served abroad have a higher prevalence of respiratory symptoms than the general population. Our hypothesis was that the risk of respiratory symptoms could be associated with exposure to a desert environment.

## Methodology

### Population

The primary study population consists of Swedish officers and soldiers who had completed at least one period of military service abroad. The control population is made up of a general population sample from four centres in Sweden.

The Swedish Armed Forces Headquarters made available the contact details of current and former soldiers and officers from selected units. They were from KS-13 to KS-16, which served in Kosovo from December 2005 to April 2008, and FS-15 to FS-17, which served in Afghanistan from April 2008 to December 2009. A random sample of those eligible was selected. Two thousand letters containing study information and instructions on how to participate were sent out to the selected addresses. Reminders were sent by postcard and information regarding the study was relayed through an article in *Fredsbaskrarna* (The Peace Beret), a veterans’ organisation magazine,[9] and on its Facebook page.[10] The data were collected primarily through a web-based system (Webropol version 2.0, Helsinki, Finland).

Among those eligible for investigation, 24 subjects had emigrated or were living under a protected identity. One had been killed in action and one had died of natural causes. Thirty-one subjects actively declined participation. A total of 1,080 (54%) were willing to take part in the study, which, after matching with the control group, left 1,032 military subjects.

### Control group

The results were compared with those of a group of individuals numbering almost 27,000, based on a cross-section of the general population, with the participants living in Stockholm, Gothenburg, Uppsala or Umeå. All the participants in this control group had previously participated in the Global Asthma and Allergy European Network (GA^2^LEN) study.[11]

### Questionnaire

The questionnaire itself was composed of two parts. The first was a copy of the GA^2^LEN questionnaire.[11] The GA^2^LEN study is a study in which data on respiratory disease and allergies have been collected from different centres in Europe. Questions of importance for this study were age, gender, smoking habits, height, weight, educational level and whether the participants had ever had asthma.

The military part of the questionnaire contained several questions about the participants’ assignments abroad, such as the number of missions abroad, the country of the assignment, whether the participant was deployed in a staff position or served more of the time in the field and whether the participant spent a considerable time in vehicles.[12]

This study was examined and approved by the regional ethics committee in Uppsala (Dnr 2011/344).

### Respiratory symptoms

The following respiratory symptoms were analysed using the GA^2^LEN questionnaire: (i) wheezing in the chest; (ii) wheezing together with breathlessness; (iii) wheezing without having a cold; (iv) nocturnal chest tightness; (v) nocturnal breathlessness (vi) and nocturnal cough.[13] The recall period for the symptoms was 12 months. In addition, we studied chronic bronchitis, which was defined as a positive answer to the question: ‘Are you used to having a cough almost every day with sputum production that lasts for at least 3 months every year during the winter?’

### Other variables

Current smoking was defined as positive answers to both questions ‘Have you ever smoked one or more cigarettes a day for more than 1 year?’ and ‘Have you smoked at all during the last month?’. The different levels of *education* were also divided into two groups; no university education and university education of at least 1 year.

### Exposure

The military participants were asked about the number of months they had served in a desert environment, whether they had had a field assignment or a staff post and whether they had been exposed to a sandstorm during the assignment.

### Statistical calculations

All the analyses were performed using STATA software, version intercooled STATA 12 (Stata Corporation, College Station, TX, USA). The prevalence of respiratory symptoms was calculated in both groups before and after matching. Each soldier was matched to one person from the reference group. The matching was performed using propensity score matching using the psmatch2 command in STATA. One-to-one matching was used and the covariates used in the matching were age, gender, smoking habits, body mass index and education level. Secondary analyses with binomial logistic regression were also made. The chi2 test and t-test were used in univariate analyses of unmatched data. A *p*-value of < 0.05 was considered statistically significant.

## Results

### Soldiers compared with the general population

Of the 1,080 soldiers, it was possible to match 1,032 to the controls from the general population sample. The characteristics of the unmatched and matched populations are presented in Table 1. The military population differed from the control population regarding age, gender distribution, body composition, education, asthma history and smoking. After matching, these differences were statistically compensated for.

**Table 1.** Characteristics of the populations (mean ± SD and %).

	Unmatched			Matched		
	Soldiers (*n* = 1,080)	Controls (*n* = 26,723)	*p*-value	Soldiers (*n* = 1,032)	Controls (*n* = 1,032)	*p*-value
Age (years)	36.1 ± 9.9	43.8 ± 16.1	<0.001	36.1 ± 9.8	35.9 ± 13.1	0.80
Women	10.6	54.7	<0.001	10.7	10.3	0.75
Height	181 ± 7	172 ± 10	<0.001	181 ± 7	180 ± 8	0.35
Weight	84 ± 13	74 ± 15	<0.001	84 ± 13	83 ± 16	0.37
University	66.6	49.9	<0.001	66.7	62.3	0.55
Ever asthma	10.1	12.6	0.01	10.0	11.7	0.18
Current smoker	6.9	14.0	<0.001	6.6	6.2	0.55

**Figure 1.**
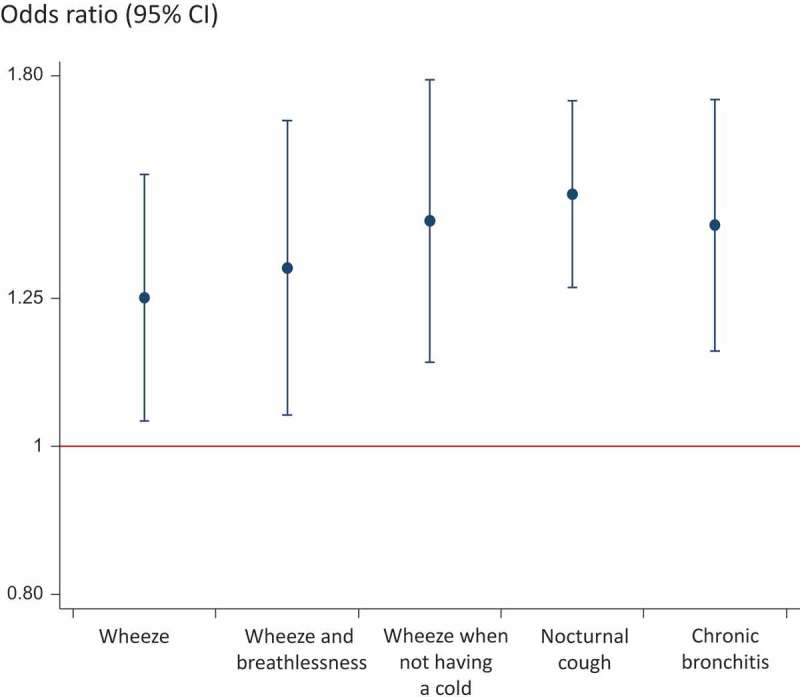
Independent association between having been on a military assignment and respiratory symptoms. (Adjusted* odds ratio (95% confidence interval.) *Adjusted for age, gender, height, weight, smoking, educational level and history of asthma

The prevalence of wheeze with breathlessness, wheeze without a cold, nocturnal cough and chronic bronchitis was significantly higher among the soldiers than the controls in the analysis using propensity score matching (Table 2). Analyses with logistic regression revealed a similar pattern, with an increased risk of wheeze, wheeze with breathlessness, wheeze when not having a cold, nocturnal cough and chronic bronchitis in the military population compared with the general population (Figure 1).

**Table 2.** Prevalence of respiratory symptoms in the soldiers and the control population before and after adjustment (%).

	Unmatched military (*n* = 1,080)	Unmatched controls (*n* = 26,723)	*p*-value	Matched military (*n* = 1,032)	Matched controls (*n* = 1,032)	*p*-value
Wheeze	16.3	15.9	0.77	16.3	13.1	0.05
Wheeze with breathlessness	9.8	9.6	0.83	9.8	7.1	0.04
Wheeze without a cold	11.1	10.4	0.41	11.1	8.1	0.03
Nocturnal chest tightness	9.6	10.7	0.25	9.6	7.2	0.06
Nocturnal breathlessness	4.2	5.3	0.11	4.2	4.1	0.91
Nocturnal cough	26.6	24.7	0.16	26.6	17.2	<0.001
Chronic bronchitis	12.4	11.3	0.32	12.3	8.2	0.003

### Analysis factors within the military group

In this group, 682 subjects had served at least part of their time in a desert environment (Afghanistan), while 393 had not. There were no significant differences in the prevalence of respiratory symptoms between these two groups.

**Figure 2.**
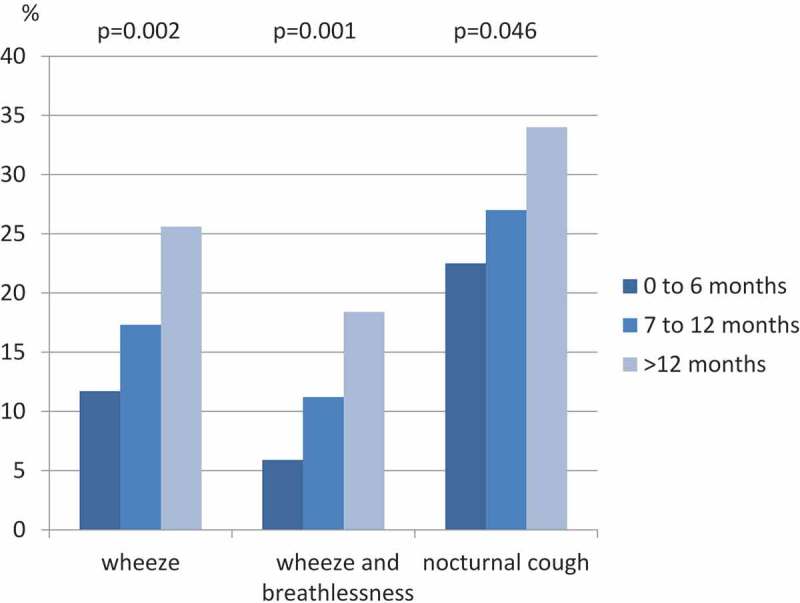
Prevalence of respiratory symptoms in association with time spent in a desert environment.

However, within the population exposed to a desert environment, there was a significant dose-response association between the months spent in this kind of environment and wheeze, wheeze and breathlessness and nocturnal cough (Figure 2). In addition, soldiers who reported having been exposed to sandstorms had a higher prevalence of nocturnal coughing (29.6 vs. 16.2%, *p* = 0.002) and chronic bronchitis (13.6 vs. 7.3%, *p* = 0.04) than those not exposed. In those with duties requiring regular transportation in vehicles, a higher prevalence of wheeze (18.1 vs. 11.4%, *p* = 0.046) and wheeze with breathlessness (11.8 vs. 5.1%, 0.02) was reported, together with a higher prevalence of nocturnal chest tightness (10.2 vs. 3.8%, *p* = 0.01), than that of the stationary soldiers, who were generally confined to camp.

## Discussion

The main findings in this study were that Swedish soldiers who had served abroad had a higher prevalence of respiratory symptoms than a matched population sample from the general population. Among soldiers who had served in a desert environment, there was as an association between exposure and the prevalence of respiratory symptoms.

To the best of our knowledge, this study is unique, as it compares respiratory health in soldiers who have served abroad with that of matched controls from the general population. Our finding of a high prevalence of respiratory symptoms among the soldiers fits in well with data from American and British studies.[7,8,14] We found that having been on a military mission abroad was related both to asthma-related symptoms such as wheeze and to cough and chronic bronchitis.

Our hypothesis was that a possible association between military assignments and negative effects on respiratory health would be related to exposure to a desert environment. It was therefore somewhat surprising that soldiers who had served in a desert environment (Afghanistan) and those that had only served in a non-desert environment (Kosovo) had the same level of respiratory symptoms. The reason for this is not clear, although it can be hypothesised that Kosovo in itself is rich in air pollution due to heavy industries, coal-burning power plants and vehicular traffic, which taken together might have a respiratory effect similar to that of a desert environment.

When focusing on the military group in this study that had desert exposure, we found significant associations regarding wheeze and nocturnal cough and the amount of time spent in a desert environment. Wheezing and nocturnal chest tightness were reported twice as frequently by the personnel that were motorised compared with those in a more stationary role. This finding may be related to the increased amounts of dust disturbed from the topsoil and stirred up into the air. Another source of inhaled airborne dust was exposure to sandstorms. Having experienced sandstorms was associated with a higher prevalence of nocturnal cough and chronic bronchitis.

An association between military assignments in a desert environment and reduced respiratory health has been reported in several previous studies.[8,15,16] A study from Kuwait showed that sandstorms have a significant impact on respiratory and asthma symptoms and hospital admissions.[17] High rates of respiratory symptoms (14%) as well as new-onset asthma among previously healthy soldiers (6.6%) were seen among soldiers post-deployment to Iraq and Afghanistan. A study from the USA examined 15,459 American soldiers deployed to Iraq or Afghanistan in 2003–2004 and found that during their deployment there was a marked increase of 69.1% among soldiers reporting respiratory illness, of which 17% reported seeking medical attention.[18]

One possible biological explanation for the associations between time spent in a desert environment and respiratory symptoms may be related to the high level of exposure to PM in a desert environment. A high level of exposure to PM is a potent factor for developing both pulmonary and cardiovascular disease.[19,20] In addition, PM-caused inflammation exacerbates pre-existing inflammatory diseases through oxidative stress and a weakened antioxidant defence.[21] Another explanation may be that psychological stress during the military assignment may lead to immunological changes [22] that may have impacted on soldiers ability to respond to airborne exposure.

The strength of this study lies in the combination of the validated GA^2^LEN study [11] as a baseline and the subsequent addition of specific dust-exposure questions. The main weakness of the study is that it is retrospective and we have no information on when the symptoms occurred in the military group. It is also possible that our methods of matching with the reference population were insufficient. The selection process that had taken place when choosing individuals that got assignments abroad would probably favour an oversampling of healthy individuals. This does not, however, explain the higher prevalence of respiratory symptoms in the military population but would rather lead to an underestimation of the actual differences. The response rate among the soldiers was 54%, which was roughly the same as in the reference group (59%). The invitation letter was sent out by the military force and we do not have any information on those that did not respond. The invitation letter made it clear that this was a study on lung health. This could have made subjects that had some kind of respiratory symptom more likely to respond. However, the invitation letter to the participants in the reference group also made it clear that they were participating in a lung health study. We therefore don’t think that this has influenced the results to large degree.

The clinical consequences of this study may be that it is important to monitor respiratory health before, during and after military deployments abroad. The study also indicates that it is important to supply protective equipment to personnel deployed in a desert environment.

We conclude that there was an association between having been on an international military assignment and respiratory symptoms and that there was also a dose-effect relationship with time spent in a desert environment and respiratory symptoms. Further research is needed to more precisely pinpoint risk areas and activities related to respiratory problems for military personal in international assignments.
